# Comprehensive characterization of endometriosis patients and disease patterns in a large clinical cohort

**DOI:** 10.1007/s00404-021-06200-w

**Published:** 2021-08-26

**Authors:** Simon Blum, Peter A. Fasching, Thomas Hildebrandt, Johannes Lermann, Felix Heindl, Tilman Born, Hannah Lubrich, Sophia Antoniadis, Karina Becker, Christine Fahlbusch, Katharina Heusinger, Stefanie Burghaus, Matthias W. Beckmann, Alexander Hein

**Affiliations:** Department of Gynecology and Obstetrics, University Endometriosis Center for Franconia, Erlangen University Hospital, Friedrich Alexander University of Erlangen-Nuremberg, Universitätsstrasse 21–23, 91054 Erlangen, Germany

**Keywords:** Endometriosis, Case–case analysis, Classification

## Abstract

**Purpose:**

In many diseases, it is possible to classify a heterogeneous group into subgroups relative to tumor biology, genetic variations, or clinical and pathological features. No such classification is available for endometriosis. In our retrospective case–case analysis we defined subgroups of endometriosis patients relative to the type and location of the endometriosis lesion and relative to basic patient characteristics.

**Methods:**

From June 2013 to July 2017, a total of 1576 patients with endometriosis diagnosed at surgery were included in this study. The patients’ history and clinical data were documented using a web-based remote data entry system. To build subgroups, all possible combinations of endometriosis locations/types (peritoneal; ovarian endometriosis; deeply infiltrating endometriosis; adenomyosis) were used. Due to the variation in group sizes, they were combined into five substantial larger groups.

**Results:**

Age, pregnancy rate, and live birth rate were identified as characteristics that significantly differed between the five patient groups that were defined. No significant differences were noted in relation to body mass index, length of menstrual cycle, age at menarche, reason for presentation, or educational level.

**Conclusion:**

This study describes basic patient characteristics in relation to common clinical subgroups in a large clinical cohort of endometriosis patients. Epidemiological information about different clinical groups may be helpful in identifying groups with specific clinical courses, potentially suggesting novel approaches to early detection and to surgical and systemic treatment.

**Supplementary Information:**

The online version contains supplementary material available at 10.1007/s00404-021-06200-w.

## Introduction

Endometriosis is a disease that is heterogeneous in relation to both symptoms and patterns of spread in the peritoneal cavity. Patients usually present with lower abdominal pain and dysmenorrhea and/or infertility, or may be asymptomatic [1–3]. In the abdomen, endometriotic lesions can be found either confined to the pelvis or spreading up to the diaphragm [4, 5]. In some patients, endometriosis lesions are not attached to the mesothelium superficially, but infiltrate adjacent structures or organs [4].

For many diseases, classification has helped to improve treatment, as it enables subgroups to be identified that have different types of risk for the most important outcomes, or different responses to specific therapies. In many diseases, it is possible to classify a heterogeneous group into subgroups relative to tumor biology, genetic variations, or clinical and pathological features [6, 7]. This can make individualized treatment possible, improving the disease-specific and overall survival for the patients. No such classification is available for endometriosis, although it is one of the most common diseases in women of reproductive age and reduces their quality of life and ability to work [8].

There are only descriptive endometriosis classifications, but they do not classify a heterogenous group into subgroups relative to clinically relevant features. The most common are specific classification systems, such as the revised American Society for Reproductive Medicine (rASRM) score [9] and the Enzian classification [10]. The latter classification system describes different locations for deeply infiltrating endometriotic lesions in general. The rASRM score is a weighted scoring system for assessing endometrial implants, plaques, endometriomas, and/or adhesions on the ovaries and/or peritoneum. None of the available classification systems gathers together all the different types of endometriotic lesion [11]. It is not possible to derive subgroups from any of these classifications that would be of meaningful diagnostic or therapeutic relevance.

Some studies have investigated the relationship between patient characteristics and endometriosis [12], but no attempts have previously been made to correlate patient characteristics such as age at diagnosis, body mass index (BMI), length of menstrual cycle, age at menarche, reason for consultation, pregnancy rates, live birth rates, or educational level with subgroups of endometriosis patients. Some of these patient characteristics have been recognized as representing risk factors for endometriosis in general [13, 14].

The aim of this study was therefore to identify groups of patients with endometriosis and to investigate the characteristics of patients in different groups. This may be helpful for further specifying and individualizing endometriosis therapy [15], whether surgical and/or medical, and thus improving the patients’ quality of life.

## Methods

### Study population

This retrospective observational study was conducted from June 2013 to July 2017 in the Department of Gynecology and Obstetrics at Erlangen University Hospital. It was designed as a case–case analysis in which different groups of patients with the same disease were compared with each other. Patients who were diagnosed with endometriosis at laparoscopy during this time span were eligible for inclusion. The time of the patient’s first operation in Erlangen University Hospital during this period was defined as the time of first presentation. A total of 1576 patients in whom endometriosis was diagnosed at surgery were identified. The diagnosis date was the earliest date on which endometriosis was first diagnosed at surgery. Of the 1576 patients, 356 had missing data or an imprecise surgical diagnosis and were excluded. A further 144 patients were excluded because information about the type and location of the endometriosis was missing. After all the exclusion criteria had been applied, the remaining study population was 1076 patients (Supplementary Fig. 1). All of the participants provided written informed consent and the medical faculty’s ethics committee approved the study.

### Data acquisition

The data collected was obtained from the patients’ charts or from a structured questionnaire completed by patients. The patients’ history and clinical data were documented using a web-based remote data entry system (electronic case report form, eCRF) [15] and were transferred to an MS Access database for further variable extraction. The eCRF collects data on 23 variables at registration, and data on at least 41 variables were collected when the patient’s medical history was being documented. In addition, at least 22 endometriosis-specific variables were recorded; if the patient underwent surgery for endometriosis, data on a further 18 variables were recorded. Detailed information was collected regarding age at first diagnosis, body mass index, age at menarche, menstrual cycle length, number of pregnancies and live births, educational level, marital status, ethnicity and current employment, diseases apart from endometriosis, reason for consultation, the way in which endometriosis was diagnosed, detailed surgical information, grade and location of endometriosis, and histological information. The basic patient characteristics are listed in Table 1. This procedure allows the type of endometriosis diagnosis to be differentiated very precisely, with the data showing whether the patient had superficial endometriosis, deeply infiltrating endometriosis (DIE), endometrioma, and/or adenomyosis uteri.

**Table 1:** Patient characteristics

Patient characteristics	Mean or frequency	Standard deviation or percent
Total	1076	100%
Age at first diagnosis (y)	33.9	± 8.4
BMI at first presentation (kg/m^2^)	24.1	± 5.1
Age at menarche (y)	13.0	± 1.5
Length of menstrual cycle (days)	28.6	± 9.0
Number of pregnancies at first presentation		
0	670	62.7%
1	190	17.8%
≥ 2	209	19.6%
Number of live births		
0	769	72.1%
1	144	13.5%
≥ 2	154	14.4%
Educational level		
University	142	36.9%
Other	243	63.1%
Ethnicity		
European	138	93.2%
Hispanic American	0	–
African	0	–
Asian	8	5.4%
Other	2	1.4%
Current employment		
Employed full-time/part-time	82	59.4%
Retired	3	2.2%
Housewife	20	14.5%
Student	28	20.3%
Unemployed	5	3.6%
Main reason for presentation		
Pain	520	48.5%
Infertility	295	27.5%
Other	258	24.0%
Previous surgery		
0	944	87.7%
1	112	10.4%
≥ 2	20	1.9%
Medical history		
No previous therapy	952	88.5%
Previous therapy	98	9.1%

The table shows the patient characteristics oft the study population

*BMI* body mass index

### Definition of groups

Endometriosis was diagnosed during laparoscopic surgery in 1076 patients. Preoperative a standardized vaginal examination and ultrasound was performed. Endometriosis was diagnosed through histological examination of the specimens. In cases where a histologic diagnosis was not available, the diagnosis and determination of the spread of endometriosis, e.g., adenomyosis, was made by the surgeon based on the preoperative and perioperative findings. The earliest date of an operation during which endometriosis was diagnosed in each patient was used as the date of diagnosis. Since no clinically relevant subgroups can be defined with the available classifications, we decided to define the subgroups based on the type and location of the endometriosis. In addition, the available classifications are too specific and do not cover all types of endometriotic lesions. To build subgroups without presuppositions, all possible combinations of endometriosis locations (peritoneal, yes/no; ovarian endometriosis, yes/no; deeply infiltrating endometriosis, yes/no; adenomyosis, yes/no) were used On the basis of these four criteria, which could either be present or not, 16 distinct groups were formed. Each patient could be assigned to a specific group. However, one group—with no endometriosis and with all four criteria negative (subgroup 16)—did not exist (Supplementary Table 1). The 1076 patients were clearly allocated to one of the 15 subgroups. The subgroups had widely varying sizes, ranging from two to 350 patients. Due to the variation in group sizes, it was not practicable to investigate all 15 subgroups and they were therefore combined into meaningful larger groups: 1—peritoneal endometriosis only; 2—peritoneal endometriosis and adenomyosis; 3—adenomyosis only; 4—peritoneal and DIE-dominant; and 5—endometrioma-dominant and other findings (Table 2 and Supplementary Fig. 2 [16]). It may be hypothesized that these different types need different treatment approaches. Further research will be needed to validate these results and attempt to identify predictive factors to assist in the choice of therapy.

**Table 2:** Division of the subgroups into five patient groups

Endometriosis location in subgroups	*n* (%) in subgroup	Group	*n* (%) in group
Peritoneal yes/endometrioma no /DIE no/adenomyosis no	350 (32.5)	Group 1: Peritoneal endometriosis only	350 (32.5)
Peritoneal yes/endometrioma no / DIE no/adenomyosis yes	142 (13.2)	Group 2: Peritoneal endometriosis and adenomyosis	142 (13.2)
Peritoneal no/endometrioma no /DIE no/adenomyosis yes	115 (10.7)	Group 3: Adenomyosis	115 (10.7)
Peritoneal yes/endometrioma no /DIE yes/adenomyosis no	105 (9.8)	Group 4: Peritoneal and DIE-dominant	275 (25.6)
Peritoneal yes/endometrioma yes/DIE yes/adenomyosis no	60 (5.6)		
Peritoneal yes/endometrioma no /DIE yes/adenomyosis yes	57 (5.3)		
Peritoneal yes/endometrioma yes/DIE yes/adenomyosis yes	53 (4.9)		
Peritoneal yes/endometrioma yes/DIE no/adenomyosis no	99 (9.2)	Group 5: Endometrioma-dominant and other	194 (18.0)
Peritoneal no/endometrioma yes /DIE no/adenomyosis no	40 (3.7)		
Peritoneal yes/endometrioma yes/DIE no/adenomyosis yes	25 (2.3)		
Peritoneal no/endometrioma no /DIE yes/adenomyosis no	14 (1.3)		
Peritoneal no/endometrioma yes /DIE no/adenomyosis yes	6 (0.6)		
Peritoneal no/endometrioma yes /DIE yes/adenomyosis no	4 (0.4)		
Peritoneal no/endometrioma yes /DIE yes/adenomyosis yes	4 (0.4)		
Peritoneal no/endometrioma no /DIE yes/adenomyosis yes	2 (0.2)		
Total	1076 (100.0)	All groups	1076 (100.0)

All the 15 subgroups were combined into meaningful larger groups

*DIE* deeply infiltrating endometriosis

### Statistical considerations

The patients’ characteristics are presented as means with standard deviation, or counts and percentages.

One-way analysis of variation (ANOVA) was performed for the characteristics of age at first diagnosis of endometriosis, BMI, age at menarche, and length of menstrual cycle. For categorical variables such as the number of pregnancies, number of live births, educational level, and main reason for presentation, a Chi-squared test was performed. Three categories were formed to examine pregnancy rates: patients without pregnancies, patients with one pregnancy, and patients with two or more pregnancies. The same procedure was used for live birth rates. Educational level was divided into two groups: patients with a university degree and patients without a university degree. The main reason for consultation was classified into pain, infertility, or other reasons.

All of the tests were two-sided, and a *P* value < 0.05 was regarded as statistically significant. Calculations were carried out using the R system for statistical computing (version 2.13.1; R Development Core Team, Vienna, Austria, 2011).

## Results

The average age at which endometriosis was first diagnosed was 33.9 years (SD ± 8.4). The study population was ethnically very homogeneous. Most of the patients were of European ethnicity (93.2%); only 5.4% were Asian, and the remainder (1.4%) were of other backgrounds. In all, 36.9% of the patients had a university degree. The patients’ mean BMI was in the upper normal range, at 24.1 (SD ± 5.1). The mean length of the menstrual cycle was 28.5 days (SD ± 9.0). The mean age at menarche was 13.0 years. The patients’ main reasons for consultation were pain (48.5%), followed by infertility (27.5%), and 24% had other reasons for consultation. A total of 944 of the 1076 patients (87.7%) underwent their first operation during this study; 112 patients (10.4%) had had one previous operation, and 20 patients (1.9%) had had more than one previous operation. Most of the patients (*n* = 952, 88.5%) had not had any previous therapy. Ninety-eight patients (9.1%) had already received drug therapy. Only 79 of them were using hormonal therapy such as contraceptives, a hormonal intrauterine device (IUD) or gonadotropin-releasing hormone (GnRH) agonists. No information about previous therapy was available for 26 patients (2.4%) (Table 1).

Peritoneal endometriosis was found in 82.8% of the 1076 patients. Diagnoses of adenomyosis uteri were less common, at 37.5%, followed by deeply infiltrating endometriosis at 27.8% and endometrioma at 27.0%.

Adenomyosis was diagnosed in 404 patients. Most often, adenomyosis was diagnosed intraoperatively. Only 76 patients had a histology of adenomyosis after hysterectomy. The 76 patients with histologically confirmed adenomyosis were almost evenly distributed among the different groups. In the group “Adenomyosis only” were 25 of 115 patients and in the group “Peritoneal endometriosis and adenomyosis” 22 of 142 patients with histologically confirmed adenomyosis. The remaining patients were distributed among the other groups. A correlation between histologically diagnosed adenomyosis and older age is possible, but due to the even distribution of the histologically confirmed adenomyosis, it is rather unlikely.

The data showed that the mean age at the first diagnosis of endometriosis differed significantly in the different groups (*P* < 0.001). Patients with peritoneal endometriosis only (group 1) were more than 3 years younger (32.5 years) than patients with adenomyosis uteri (35.9 years) (Table 3; Supplementary Fig. 3). In comparison with the other groups, the group with adenomyosis only had the lowest rate without pregnancy (42.5%) and the lowest rate without a live birth (53.6%). Over 30.4% of patients with adenomyosis had at least two live births, while 16.1% had one live birth. This means that 46.5% of the patients with adenomyosis had at least one child, in comparison with the other groups, in which fewer than 30% had at least one live birth (Supplementary Figs. 4 and 5). There were also significant results for pregnancies (*P* < 0.001) and for live births (*P* < 0.001) (Table 3). In view of the high rates of pregnancy and live births, infertility as the main reason for consultation was rare in the group with adenomyosis. However, there were no significant differences between the five groups in relation to this parameter (*P* = 0.62). There were also no significant differences between the groups in relation to body mass index, educational level, length of menstrual cycle, or age at menarche.

**Table 3:** Patient characteristics in the five patient groups

	Group 1 Peritoneal only *n* = 350 (32.5%) Mean (SD) or *n* (%)	Group 2 Peritoneal & adenomyosis *n* = 142 (13.2%) Mean (SD) or *n* (%)	Group 3 Adenomyosis only *n* = 115 (10.7%) Mean (SD) or *n* (%)	Group 4 Peritoneal & DIE *n* = 275 (25.6%) Mean (SD) or *n* (%)	Group 5 Endometrioma *n* = 194 (18.0%) Mean (SD) or *n* (%)	*Total* *n* = 1076 (100%) Mean (SD) or *n* (%)
Age at first diagnosis (y)	32.6 (9.1) *n* = 350	33.3 (8.1) *n* = 142	35.9 (9.9) *n* = 115	34.3 (7.2) *n* = 275	35.0 (7.5) *n* = 194	33.9 (8.4) *n* = 1076
BMI at first presentation (kg/m^2^)	24.0 (5.5) *n* = 279	24.5 (5.1) *n* = 90	24.3 (4.7) *n* = 74	24.0 (5.2) *n* = 211	24.2 (4.7) *n* = 159	24.1 (5.1) *n* = 813
Age at menarche (y)	13.0 (1.4) *n* = 318	13.0 (1.7) *n* = 137	12.8 (1.6) *n* = 106	13.1 (1.4) *n* = 255	13.0 (1.5) *n* = 175	13.0 (1.5) *n* = 991
Length of menstrual cycle (days)	28.1 (5.2) *n* = 183	29.1 (8.5) *n* = 80	30.4 (14.2) *n* = 58	27.7 (3.3) *n* = 168	29.4 (15.0) *n* = 111	28.6 (9.0) *n* = 600
No. of pregnancies at first presentation						
0	229 (66.2)	82 (58.2)	48 (42.5)	186 (67.6)	125 (64.4)	670 (62.7)
1	55 (15.9)	34 (24.1)	20 (17.7)	51 (18.5)	30 (15.5)	190 (17.8)
≥ 2	62 (17.9)	25 (17.7)	45 (39.8)	38 (13.8)	39 (20.1)	209 (19.6)
Total	346 (100)	141 (100)	113 (100)	275 (100)	194 (100)	1069 (100)
No. of live births						
0	259 (75.1)	102 (72.3)	60 (53.6)	211 (76.7)	137 (70.6)	769 (72.1)
1	44 (12.8)	18 (12.8)	18 (16.1)	36 (13.1)	28 (14.4)	144 (13.5)
≥ 2	42 (12.2)	21 (14.9)	34 (30.4)	28 (10.2)	29 (14.9)	154 (14.4)
Total	345 (100)	141 (100)	112 (100)	275 (100)	194 (100)	1067 (100)
Educational level						
University	52 (39.4)	15 (34.1)	8 (26.7)	42 (40.4)	25 (33.3)	142 (36.9)
Other	80 (60.6)	29 (65.9)	22 (73.3)	62 (59.6)	50 (66.6)	243 (63.1)
Total	132 (100)	44 (100)	30 (100)	104 (100)	75 (100)	385 (100)
Ethnicity						
European	43 (91.5)	16 (94.1)	8 (88.9)	44 (95.7)	27 (93.1)	138 (93.2)
Hispanic American	0 (0.0)	0 (0.0)	0 (0.0)	0 (0.0)	0 (0.0)	0 (0.0)
African	0 (0.0)	0 (0.0)	0 (0.0)	0 (0.0)	0 (0.0)	0 (0.0)
Asian	3 (6.4)	1 (5.9)	0 (0.0)	2 (4.3)	2 (6.9)	8 (5.4)
Other	1 (2.1)	0 (0.0)	1 (11.1)	0 (0.0)	0 (0.0)	2 (1.4)
Total	47 (100)	17 (100)	9 (100)	46 (100)	29 (100)	148 (100)
Current employment						
Employed full-time/part-time	22 (50.0)	10 (71.4)	7 (50.0)	27 (71.1)	16 (57.1)	82 (59.4)
Retired	2 (4.5)	0 (0.0)	0 (0.0)	0 (0.0)	1 (3.6)	3 (2.2)
Housewife	6 (13.6)	1 (7.1)	4 (28.6)	3 (7.9)	6 (21.4)	20 (14.5)
Student	13 (29.5)	3 (21.4)	2 (14.3)	7 (18.4)	3 (10.7)	28 (20.3)
Unemployed	1 (2.3)	0 (0.0)	1 (7.1)	1 (2.6)	2 (7.1)	5 (3.6)
Total	44 (100)	14 (100)	14 (100)	38 (100)	28 (100)	138 (100)
Main reason for presentation						
Pain	166 (47.7)	73 (51.4)	67 (58.3)	129 (46.9)	85 (44.0)	520 (48.5)
Infertility	87 (25.0)	45 (31.7)	24 (20.9)	85 (30.9)	54 (28.0)	295 (27.5)
Other	95 (27.3)	24 (16.9)	24 (20.9)	61 (22.2)	54 (28.0)	258 (24.0)
Total	348 (100)	142 (100)	115 (100)	275 (100)	193 (100)	1073 (100)

The table shows the patient characteristics per group. In our study we tested for differences between the five groups. The mean age at first diagnosis of endometriosis differed significantly in the different groups (*P* < 0.001). There were also significant results for pregnancies (*P* < 0.001) and for live births (*P* < 0.001). There were no significant differences between the other patient characteristics

*BMI* body mass index, *DIE* deeply infiltrating endometriosis

## Discussion

In this retrospective case–case study, basic patient characteristics were associated with the location of endometriosis by developing subgroups of endometriosis lesions. In five defined groups, significant differences were found in the patient’s mean ages at first diagnosis of endometriosis. Significant differences were also found between pregnancy rates and also between live birth rates in the groups.

It has been shown in previous studies that there is a risk of developing endometriosis only during women’s reproductive years. In earlier studies, the highest risk was found to be at 44 years of age [17], with another peak between 25 and 29 years. The highest risk reported in recent studies was between 25 and 29 years. The risk was found to decrease after the age of 44 in all of the studies [12, 18, 19]. In the present study, the mean age at onset was 33.9 years. There is a known delay between the first symptoms and the diagnosis of endometriosis at surgery. In addition, the time of the first operation is influenced by socio–economic factors such as education, access to special medical facilities, or age at the onset of symptoms [8, 20, 21]. This can delay the surgical diagnosis of endometriosis. However, as the present study involved a case–case analysis, the mean age may be influenced, but not the differences between the groups.

There have only been a few studies examining endometriosis groups. In one study, no significant difference was found between patients with DIE and patients with peritoneal endometriosis or endometrioma [22]. In another study, a significant difference was found between the average age of women diagnosed with endometriosis and women diagnosed with adenomyosis uteri. Patients with endometriosis were younger than those with adenomyosis uteri [23]. The present study examined whether there were any differences between five defined groups. The mean age at first diagnosis of endometriosis differed significantly in the group analysis. Patients with adenomyosis uteri were 3 years older than patients with peritoneal endometriosis only. In the present study, there were two diagnostic methods of identifying adenomyosis uteri—either through histology or the surgeon’s impression. However, hysterectomy was avoided in young patients, and this may have influenced the high mean age in patients with adenomyosis uteri. Nevertheless, the mean age in the group with peritoneal endometriosis and adenomyosis uteri was the second lowest in all the groups. It is not therefore expected that the results may have been biased as a result of the diagnostic methods used.

The data showed significant differences between the groups in relation to pregnancy rates and also live birth rates, and suggest that patients with adenomyosis have a higher pregnancy rate and live birth rate than patients in the other four groups. In previous studies, adenomyosis in particular is considered to be a cause of infertility and miscarriage when assisted reproductive techniques are used [24, 25]. Other studies have primarily examined the influence of endometriosis on infertility [26]. However, there are no data on the pregnancy rate and live birth rate with spontaneous conception. In addition, no studies were found in the literature that have compared pregnancy rates or live birth rates between different groups with endometriosis. The average rates for women without pregnancy and without live births were high. However, comparison with a control group was not available and it is therefore not possible to draw any conclusions regarding infertility in comparison with a healthy population.

The odds of developing endometriosis have been reported to be lower among women with histologically confirmed endometriosis who had a large versus lean body size [27]; a higher body mass index may also be associated with a lower risk of endometriosis [28]. In a subgroup analysis, obese women were found to be more likely to have a surgical diagnosis of adenomyosis [23]. In the present study, the groups with adenomyosis uteri had the highest BMI, but the differences were not significant.

With regard to the menstrual cycle, current studies assume that frequent menstrual bleeding is a risk factor for endometriosis. Early age at menarche, shorter menstrual cycles and thus more frequent period bleeding, longer bleeding periods, and few pregnancies appear to be risk factors [29]. It has previously been reported that age at menarche did not differ between patients who underwent hysterectomy with a diagnosis of adenomyosis and those who did not have a diagnosis of adenomyosis [30].

There is not known to be any association between endometriosis and educational level [8], but no subgroup analyses have as yet been published.

No differences were found between the groups with regard to the main reasons for consultation. These data are consistent with data showing that there is no association between the extent of endometriosis and pain [31]. On the other hand, there is some evidence that adenomyosis uteri is associated with pain [32].

Case selection in a hospital-based study may be biased by the variety of health care that is provided in the hospital concerned. Women seeking help for pelvic pain might increase the numbers of cases of pain-inducing endometriosis, and a hospital providing specialized health care for infertility patients might have larger numbers of patients. In the present study, the hospital is a center for all types of treatment, so that a bias toward one of these groups seems unlikely. Patients who underwent surgical therapy were selected. The study group included therefore does not correspond to the normal distribution of endometriosis patients and thus does not represent an adequate epidemiological picture.

One major limitation of the study is that pregnancy rates and live birth rates may be influenced by other patient characteristics, such as the main reason for consultation or mean age. The present data are only able to show associations, but not causality.

Another limitation is the type of diagnostic method used to identify adenomyosis uteri through clinical aspects and the surgeon’s impression. The diagnosis of adenomyosis uteri is clinically challenging, as there is no consensus on the best imaging features for a nonsurgical diagnosis of adenomyosis [33]. Nevertheless, standardized preoperative ultrasound was performed in our study in 1045 of the 1076 cases. Adenomyosis was suspected in 135 patients. All of the patients underwent a preoperative vaginal examination. The gold standard of hysterectomy cannot be applied in this population. In relation to international guidelines, it was decided that the surgeon should make the intraoperative diagnosis of an endometriosis on the basis of the medical history and preoperative clinical findings [34]. Histological diagnosis of the disease or longer-term follow-up data might be helpful for refining the groups.

In conclusion, this study has added to the evidence that different subgroups exist among endometriosis patients. It may be hypothesized that these different types need different treatment approaches. Further research will be needed to confirm these results and attempt to identify predictive factors to assist in the choice of therapy,

## Supplementary Information

Below is the link to the electronic supplementary material.

Supplementary file1 (DOCX 258 KB)
