# Supplementary material for: Comprehensive characterization of endometriosis patients and disease patterns in a large clinical cohort
Source: Arch Gynecol Obstet. 2021 Aug 26;305(4):977–84. doi: 10.1007/s00404-021-06200-w (PMC8967757; doi:10.1007/s00404-021-06200-w)
Supplement: Supplementary file 1 — Supplementary file1 (DOCX 258 KB) [file 404_2021_6200_MOESM1_ESM.docx]

**SUPPLEMENTARY TABLE 1
Division into subgroups of endometriosis patients.**

| **Subgroup** | **Endometriosis location** | | | |
| --- | --- | --- | --- | --- |
|  | **Peritoneal** | **Endometrioma** | **DIE** | **Adenomyosis** |
| 1 | Yes | Yes | Yes | Yes |
| 2 | Yes | Yes | Yes | No |
| 3 | Yes | Yes | No | Yes |
| 4 | Yes | Yes | No | No |
| 5 | Yes | No | Yes | Yes |
| 6 | Yes | No | Yes | No |
| 7 | Yes | No | No | Yes |
| 8 | Yes | No | No | No |
| 9 | No | Yes | Yes | Yes |
| 10 | No | Yes | Yes | No |
| 11 | No | Yes | No | Yes |
| 12 | No | Yes | No | No |
| 13 | No | No | Yes | Yes |
| 14 | No | No | Yes | No |
| 15 | No | No | No | Yes |
| 16 | No | No | No | No |

To build subgroups, all possible combinations of endometriosis locations (peritoneal, yes/no; ovarian endometriosis, yes/no; deeply infiltrating endometriosis, yes/no; adenomyosis, yes/no) were used.

DIE, deeply infiltrating endometriosis.

**SUPPLEMENTARY FIGURE 1
Flow chart of patients included.**
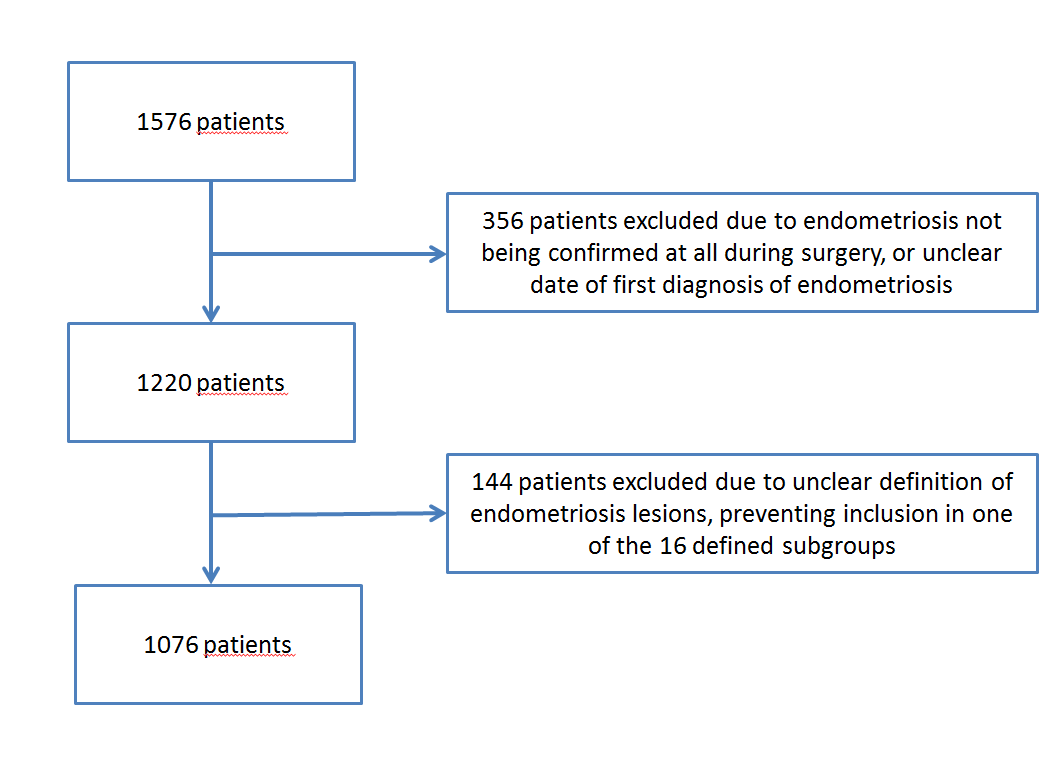

Of the 1576 patients enrolled in whom endometriosis was diagnosed at surgery, 356 had missing data or an imprecise surgical diagnosis and were excluded. A further 144 patients were excluded because information about the type and location of the endometriosis was missing.

**SUPPLEMENTARY FIGURE 2
Area-proportional Venn diagram of the groups.***

**
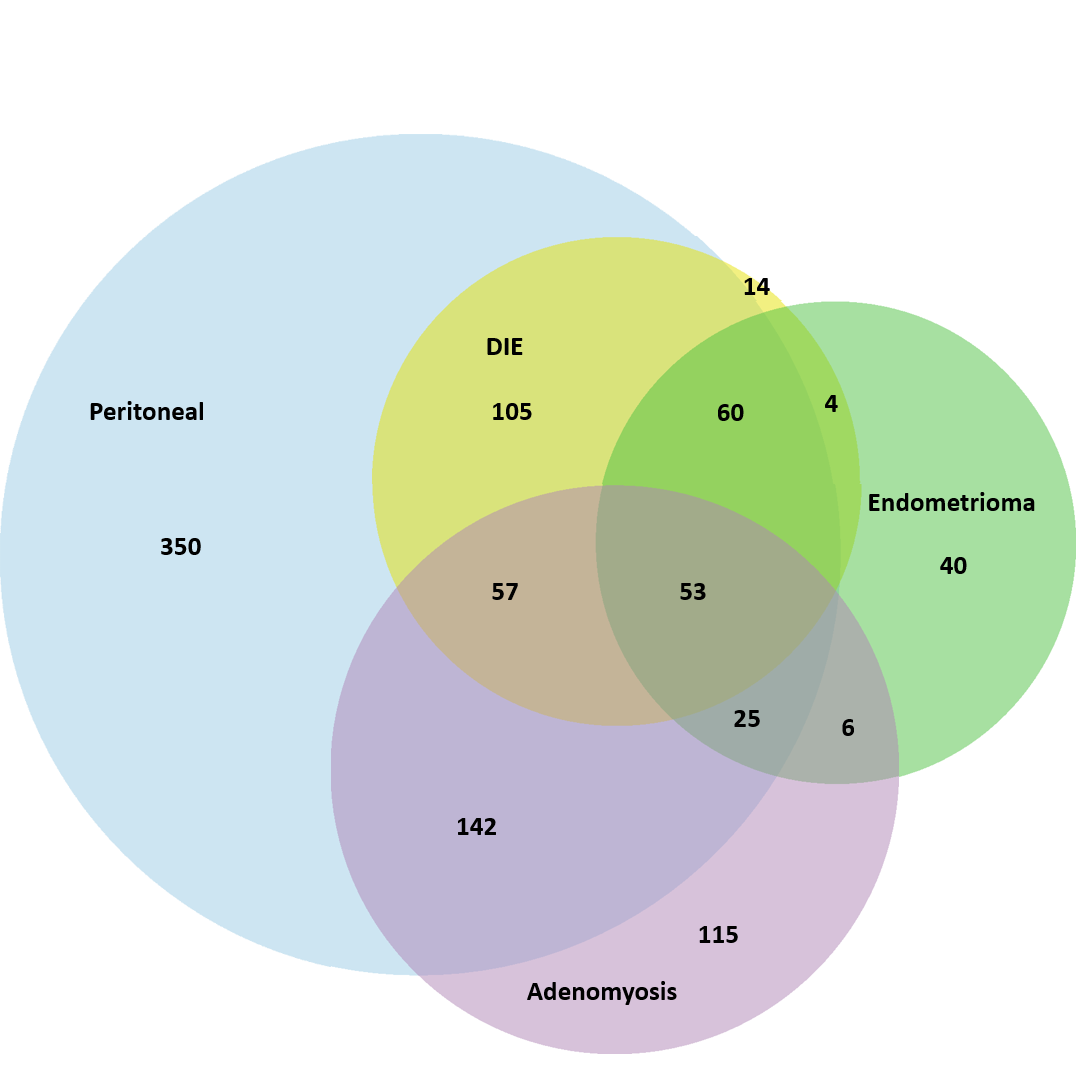
**

The Figure shows the distribution of all 1076 patients according to the four criteria (peritoneal endometriosis, endometrioma, deep infiltrating endometriosis, adenomyosis).
* Due to mathematical reasons (more degrees of freedom are needed) the overlap between more than two circles cannot always correspond exactly with the number of IDs it represents.

DIE, deeply infiltrating endometriosis.

**SUPPLEMENTARY FIGURE 3
Age at first diagnosis.**


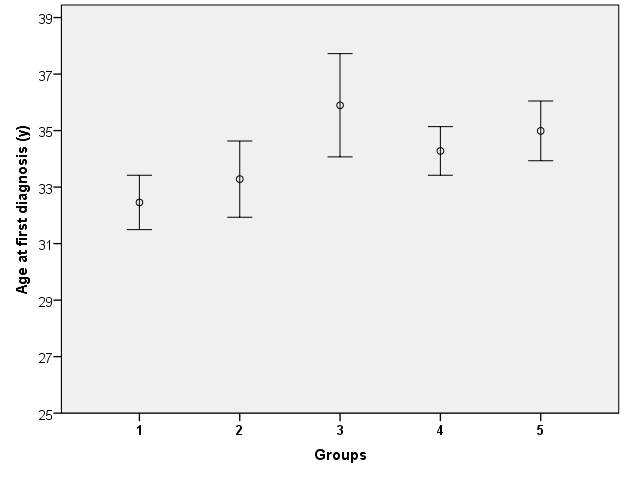


The analysis included all enrolled patients. The mean age at first diagnosis in years and the 95 % CI for each of the five groups (1, peritoneal endometriosis only; 2, peritoneal endometriosis and adenomyosis; 3, adenomyosis only; 4, peritoneal and DIE-dominant; and 5, endometrioma-dominant) is shown.

**SUPPLEMENTARY FIGURE 4
Pregnancies at first presentation.**


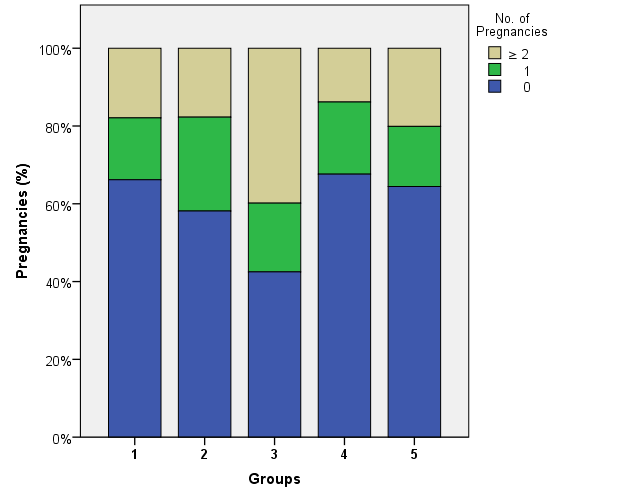


The analysis included all patients with known pregnancy and birth rates. The diagram shows the percentage distribution of the pregnancy rates within the five groups (1, peritoneal endometriosis only; 2, peritoneal endometriosis and adenomyosis; 3, adenomyosis only; 4, peritoneal and DIE-dominant; and 5, endometrioma-dominant). The pregnancy rates were grouped into three categories (≥ 2 pregnancies, 1 pregnancy, without pregnancy).

**SUPPLEMENTARY FIGURE 5
Live births at first presentation.**


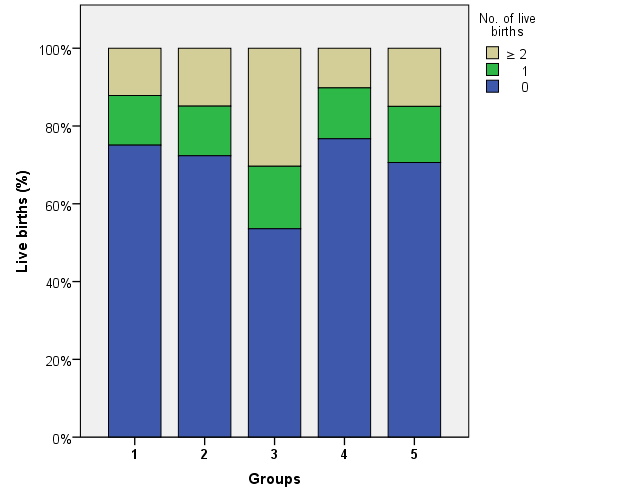


The analysis included all patients with known pregnancy and birth rates. The diagram shows the percentage distribution of the live birth rates within the five groups (1, peritoneal endometriosis only; 2, peritoneal endometriosis and adenomyosis; 3, adenomyosis only; 4, peritoneal and DIE-dominant; and 5, endometrioma-dominant). The live birth rates were grouped into three categories (≥ 2 live births, 1 live birth, without live birth).
